# Resultant equations for training load monitoring during a standard microcycle in sub-elite youth football: a principal components approach

**DOI:** 10.7717/peerj.15806

**Published:** 2023-08-04

**Authors:** José Eduardo Teixeira, Pedro Forte, Ricardo Ferraz, Luís Branquinho, Ryland Morgans, António José Silva, António Miguel Monteiro, Tiago M. Barbosa

**Affiliations:** 1 Research Centre in Sports, Health and Human Development, Covilhã, Portugal; 2 Department of Sport Sciences, Instituto Politécnico de Bragança, Bragança, Portugal; 3 Department of Sport Sciences, Polytechnic Institute of Guarda, Guarda, Portugal; 4 CI-ISCE Douro, Higher Institute of Educational Sciences of the Douro, Penafiel, Portugal; 5 Department of Sport Sciences, University of Beira Interior, Covilhã, Portugal; 6 Institute for Coaching and Performance, University of Central Lancashire, Preston, United Kingdom; 7 Sport Sciences, University of Trás-os-Montes and Alto Douro, Vila Real, Portugal

**Keywords:** Youth, Workload, Soccer, Global positioning system, PCA

## Abstract

Applying data-reduction techniques to extract meaningful information from electronic performance and tracking systems (EPTS) has become a hot topic in football training load (TL) monitoring. The aim of this study was to reduce the dimensionality of the internal and external load measures, by a principal component approach, to describe and explain the resultant equations for TL monitoring during a standard in-season microcycle in sub-elite youth football. Additionally, it is intended to identify the most representative measure for each principal component. A principal component analysis (PCA) was conducted with a Monte Carlo parallel analysis and VariMax rotation to extract baseline characteristics, external TL, heart rate (HR)-based measures and perceived exertion. Training data were collected from sixty sub-elite young football players during a 6-week training period using 18 Hz global positioning system (GPS) with inertial sensors, 1 Hz short-range telemetry system, total quality recovery (TQR) and rating of perceived exertion (RPE). Five principal components accounted for 68.7% of the total variance explained in the training data. Resultant equations from PCA was subdivided into: (1) explosiveness, accelerations and impacts (27.4%); (2) high-speed running (16.2%); (3) HR-based measures (10.0%); (4) baseline characteristics (8.3%); and (5) average running velocity (6.7%). Considering the highest factor in each principal component, decelerations (PCA 1), sprint distance (PCA 2), average HR (PCA 3), chronological age (PCA 4) and maximal speed (PCA 5) are the conditional dimension to be considered in TL monitoring during a standard microcycle in sub-elite youth football players. Current research provides the first composite equations to extract the most representative components during a standard in-season microcycle in sub-elite youth football players. Futures research should expand the resultant equations within training days, by considering other well-being measures, technical-tactical skills and match-related contextual factors.

## Introduction

Training load (TL) monitoring has become a research hot topic in youth football (Impellizzeri et al., 2022; Staunton et al., 2021). This is largely due to the growing access to electronic performance and tracking systems (EPTS) that provides valid TL measures (de Dios-Álvarez et al., 2021; Oliva-Lozano & Muyor, 2022). In recent years, the weekly TL variation has been extensively analyzed in elite and sub-elite football contexts (Teixeira et al., 2022a). Training monitoring has been extensively performed using objective and subjective methods to monitor internal training load (ITL) and external training load (ETL) (Impellizzeri et al., 2022). Global positioning system (GPS) devices have become a customary, low-cost and optimal navigation satellite system to extract valid and reliable ETL outcomes (*e.g*., distances, sprints, accelerations (ACC), change of directions or body impacts) (Beato et al., 2018; Buchheit et al., 2021). Otherwise, the ITL has been usually monitored by heart rate (HR) and perceived exertion using non-invasive wearable sensor systems, rating perceived exertion (RPE) and total quality recovery (TQR) scales (Haddad et al., 2017; Brink et al., 2010). The research has shown a significant correlation between ETL and ITL in young athletes, however it is still difficult to interpret fitness-recovery status (Impellizzeri et al., 2022). Combining ETL and ITL has been reported as a valid strategy to analyse dose-response dissonances, however the major influencing factor remain to be defined (Bourdon et al., 2017; Teixeira et al., 2021a).

Additionally, the emergent tracking tools appears to have created confusion in dose-response considerations given the data analysis requirement to extract relevant information from large amounts of data (Griffin et al., 2021; Scantlebury et al., 2020). This kind of tracking device can provide big datasets express as a thousand data per second expressed by a large number of variables depending on the time-motion technology used (Rojas-Valverde et al., 2020; Ruan et al., 2022). Otherwise, another challenge has been to standardize the physical and psychophysiological data in meaningful information (Impellizzeri et al., 2022; Staunton et al., 2021; Vanrenterghem et al., 2017). As well, capturing the training frequency, intensity, time/duration, type, volume, and progression (FITT-VP) variables is another critical challenge created by tracking systems (Staunton et al., 2021; Scantlebury et al., 2020). Thus, it is more critical than ever to turning datasets into relevant information for athlete-monitoring cycle (Teixeira et al., 2021a; Weaving et al., 2019). Afterwards, the data-reduction techniques has been applied to explain the dimensionality of the TL variables in different football codes such as futsal (Rico-González et al., 2022a), Australian football (Sheehan et al., 2020), rugby (Scantlebury et al., 2020; Weaving et al., 2020) and Gaelic football (Gamble et al., 2019).

Principal component analysis (PCA) is one of the most used data-reduction techniques to extract redundant information from TL data in football (Rico-González et al., 2022b; Rojas-Valverde et al., 2020). Using a PCA approach, a significant percentage of the total variance in a dataset can be extracted (Warmenhoven et al., 2019). Thus, PCA analysis allows to reduce the complexity in a large group of correlated variables by determining the principal components (O’Donoghue, 2008; Rojas-Valverde et al., 2020). Recently, a systematic review conducted in football reported a 77.1% of explained variance in 12.8 extracted variables out of 51.4 variables distributed over 6.4 principal components (Rojas-Valverde et al., 2020). However, the studies with PCA approaches has focused mainly on TL monitoring in professional and elite youth football (Casamichana et al., 2019; Scantlebury et al., 2020; Sheehan et al., 2020). Until now, PCA approaches were only applied in elite football contexts to simplify the TL having regard to different game formats (Casamichana et al., 2019; Zurutuza et al., 2020), contextual factors (Gonçalves et al., 2019; Oliva-Lozano et al., 2021), competition level (Ricotti et al., 2013), positional role (Moura et al., 2015), tactical behaviour (Ric et al., 2016; Rico-González et al., 2022b) and motor skills (Los Arcos, Mendiguchia & Javier, 2017). Recently, some studies have described the application of TL monitoring strategies during a weekly microcycle in sub-elite youth football, expressing by a low seasonal variation and a high weekly variation (Teixeira et al., 2021b, 2022b). Therefore, it is important to establish the major influencing factor for an accurate training monitoring and manipulation during a standard microcyle. Also, an optical TL monitoring can enhance a proper long-term athlete development, injury prevention and training design (Pino-Ortega et al., 2021; Rico-González et al., 2022c; Rojas-Valverde et al., 2020). More specifically, this can help research, practitioners and coaches to prescribe adequate training intensity over a standard microcycle in youth football (Rico-González et al., 2022a). Therefore it is critical to standardize and reduce the dimensionality of the weekly training data in young football players from sub-elite contexts (Teixeira et al., 2022c; Trecroci et al., 2018). Thus, the aim of this study was to reduce the dimensionality of the internal and external load measures, by a PCA approach, in order to describe and explain the resultant equations for TL monitoring during a standard microcycle in a sub-elite youth football players. Additionally, it is intended to identify the most representative measure for each principal component.

## Methods

### Participants

Sixty sub-elite youth and male football players were included this study from an under (U) 15 (*n* = 20), U17 (*n* = 20) and U19 (*n* = 20) sub-elite youth football academy (Table 1). All parents or legal guardians were written briefed about research aims and risks, providing a written consent for participant’s inclusion. The research was developed in accordance with the Declaration of Helsinki (Winter & Maughan, 2009) with an ethical approval from the local Ethical Committee from the University of Trás-os-Montes e Alto Douro (3379-5002PA67807).

**Table 1:** Description baseline characteristics of participants.

Variables	U15 (*n* = 20)	U17 (*n* = 20)	U19 (*n* = 20)	Overall (*n* = 60)
Age (y)	13.28 ± 0.49	15.39 ± 0.51	17.29 ± 0.55	15.19 ± 1.75
RA (a.u.)	0.25 ± 0.17	0.25 ± 0.17	0.24 ± 0.20	0.25 ± 0.18
MO (a.u.)	−0.42 ± 0.76	2.02 ± 1.09	2.23 ± 1.49	1.33 ± 1.67
Height (m)	1.69 ± 0.78	1.76 ± 0.48	1.76 ± 0.70	1.74 ± 0.08
Weight (kg)	55.67 ± 9.41	64.28 ± 6.61	68.90 ± 8.39	62.48 ± 10.03
BMI (kg/m^2^)	19.29 ± 1.99	20.68 ± 1.79	22.11 ± 1.50	20.61 ± 2.14
Sitting height (cm)	81.96 ± 5.78	92.02 ± 7.61	90.73 ± 8.06	88.36 ± 8.51
PHV (cm)	14.18 ± 0.80	13.90 ± 1.09	14.46 ± 1.87	14.20 ± 1.39
Experience (y)	4.82 ± 0.90	6.64 ± 1.65	8.81 ± 1.70	6.76 ± 1.42

**Note:**

Abbreviations: a.u., arbitrary unit; BMI, body mass index; MO, maturity offset; PHV, peak high velocity; RA, relative age; y, years.

### Quasi-experimental approach

**Figure 1:**
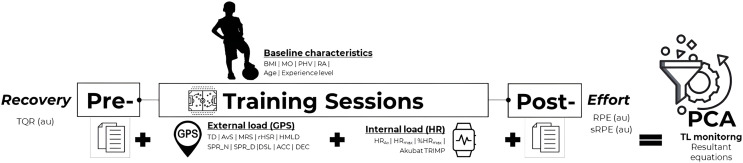
Training load monitoring using a prospective, observational and cross-sectional design.

Current research has a prospective, observational and cross-sectional design, by applying an individual TL strategy *via* GPS technology, HR monitoring system, RPE and TQR scales. Resultant equations for TL monitoring in sub-elite youth football was obtained by a PCA approach. The weekly TL was continuously monitored during 2019–2020 in-season, representing a total of 6-week period from 18 training sessions and 324 observation cases (Teixeira et al., 2021b, 2022d). A minimum of 150 observation cases (*i.e*., 5 to 10 cases per variable) was assured to perform PCA analysis (Jolliffe & Cadima, 2016). Figure 1 summarizes the procedures for quasi-experimental approach.

### Procedures

The training data eligibility considered the following inclusion criteria: (a) youth football players aged between 13 and 20 years old (*i.e*., U15, U17 and U19) (Teixeira et al., 2021a); (b) young football players should have at least 5 years of competitive experience in football (Ford et al., 2020); (c) training data featured at least 35 consecutive playing minutes without any break for injury, abandonment or other arbitrary reason (de Dios-Álvarez et al., 2021); (d) training data considered a competitive one-game week schedule and three training sessions per week (Teixeira et al., 2021b, 2022a). The exclusion of cases occurred when the following exclusion criteria were met: (a) event of absence, injury, illness and abandonment during monitored training sessions; (b) players that were not integrated in the common team session due to rehabilitation, complementary and/or individual training sessions; (c) the match data was not included in the analysis (Teixeira et al., 2022d). For ETL and ITL monitoring, each participant wore the micro-technology (*i.e*., GPS and HR) within a little pocket on the upper back between both scapulae of a custom-made vest (Beato et al., 2018). All methodological procedures for ETL and ITL were previously applied for 2 weeks to familiarize players with data collection (de Dios-Álvarez et al., 2021).

Using a “match day minus format” (MD), the weekly microcycle included the training sessions MD-3 (Tuesday), MD-2 (Wednesday), and MD-1 (Friday). The number of observation for each training day was: MD-3 (*n* = 41), MD-2 (*n* = 38), and MD-1 (*n* = 44) (Teixeira et al., 2022d, 2021b). The training days for the three age groups were the same following this order: U15—6 to 7:30 PM; U17—7:30 to 9:00 PM; U19—9:00 PM to 10:30 PM. The average duration of training sessions had the following lengths for each age group: U15 = 148.99 min; U17 = 132.46 min; U19 = 195.95 min. Medical and logistical staff members ensured that all training classes had standardized clothes, nutrition and medical care during training sessions (Teixeira et al., 2022d). All training sessions were performed on a synthetic turf outdoor pitch with official dimensions (FIFA standard; 100 m × 70 m) and similar environment conditions (*i.e*., 14–20 °C; relative humidity 52–66%) (Coutinho et al., 2015).

### Weekly standard microcycle

Table 2 showed the weekly training overview in the studied sub-elite youth football academy. The standard microcycle was planned in accordance with the following key points: (i) training aims, time duration and pitch dimensions; (ii) physiological target and speed, agility and quickness (SAQ) emphasis; (iv) training tasks and exercises. Weekly training overview was designed according to field notes and academy training model. Also, current typical microcycle was designed during an in-season standard microcycle with aforementioned training days (*i.e*., MD-3, MD-2 and MD-1) (Branquinho, Ferraz & Marques, 2021; Rago et al., 2020). Small, medium, large-sided, and simulated games (*i.e*., SSG, MSG, LSG) was categorized in accordance with Zurutuza et al. (2020). The SAQ training was classified by Trecroci et al. (2016) for sub-elite football players.

**Table 2:** Weekly standard microcycle in the sampled sub-elite youth football academy.

Construct	MD-3 (Tuesday)	MD-2 (Wednesday)	MD-1 (Friday)
Aim (tactical)	Recovery/technical skills	Acquisitive training focused on game principles (collective behaviour and organization)	Finishing situations and tactical schemes
Duration	90 min	90 min	90 min
Dimensions	50 m × 60 m (half field)	100 m × 60 m (entire field)	50 m × 60 m (half field)
Physiological set	75–80% HR_max_	90–95% HR_max_	>85% MRS
SAQ	Strength (Quickness, COD and agility)	Endurance/Aerobic	Speed
Warm up	Technical and coordination skills	Dynamic stretching	Plyometric exercises and SSC
Training tasks	(1) SSG, MSG, and ball possession (small areas);	(1) Ball possession, LSG and simulated games;	(1) Finishing exercises (*i.e*., individual, sectional and intersecional situations: 1 × 0 + GK to 11 × 0 + GK);
	(2) Individual enrichment training (*i.e*., 1v1 to 3v3).	(2) Game strategy.	(2) Tactical schemes (*i.e*., outsides and corners).

**Note:**

Abbreviations: COD, change of direction speed; GK, goalkeeper; HR_max_, maximal heart rate; LSG, large-side games; MD, “match day minus” format; MSG, medium-sided games; MRS, maximum running speed; PHV, peak high velocity; SAQ, speed, agility and quickness; SSC, stretch-shortening cycle; SSG, small-sided games.

### Training load measures

Table 3 described the construct, measurement unit, and formula for each ETL and ITL measure. All constructs were considered according to previous TL-based reports, specifically: (i) total distance (TD); (ii) average running velocity; (iii) high-speed running (HSR); (iv) explosiveness, ACC and body impacts; (v) HR-based measures; and (vi) perceived exertion and recovery (Rico-González et al., 2022a; Sheehan et al., 2020; Teixeira et al., 2021a).

**Table 3:** Construct, description and formulas from external and internal training load.

TL	Constructs	Variable	Description and formula
ETL	Total distance	TD (m)	Total distance covered (in meters)
	Average running velocity	AvS (m·min^−1^)	Game pace or average speed distance in meter per minutes.
		MRS (m·s^−1^)	Maximal speed in meter per seconds
	High intensity running	rHSR (m)	Relative high-speed running (rHSR) distance (m) covered at 19.8–25.1 km·h^−1^.
		SPR (n | m)	The sprints were measured by number and average sprint distance (m) in a velocity >25.1 km·h^−1^.
	Explosiveness, accelerations and impacts	HMLD (m)	High metabolic load distance (HMLD) is a metabolic variable defined as the distance, expressed in meters, covered by player when the metabolic power exceeds 25.5 W·kg^−1^.
		DSL (au)	The DSL was computed by measuring the sum of the accelerations in the three orthogonal axes of movement (expressed as a G force > 2G).
		ACC | DEC (m·s^−2^)	Number of accelerations (>3 m·s^−2^) and decelerations.
ITL	HR	HR_max_ (bpm)	Maximum heart rate (HR_max_)
		AvHR (bpm)	Average heart rate (AvHR).
		%HR_max_	Percentage of HR_max_ (%HR_max_)
		TRIMP (au)	Akubat TRIMP (iTRIMP) = Training duration × 0.2053e3.5179x. Among which e = Napierian logarithms, 3.5179 is the exponent, and x = HRratio.
	Perceived exertion and recovery	RPE (au)	Perceived exertion was measured by 15-point Portuguese Borg Rating of Perceived Exertion 6–20 Scale (Borg RPE 6–20).
		sRPE	The sRPE was obtained by multiplying total duration of training sessions for each individual RPE score.
		TQR (au)	To monitor recovery, each player was asked to report the TQR score on a scale from 6 to 20.

**Note:**

Abbreviations: ACC, acceleration; AvHR, average heart rate; AvS, average speed; DEC, deceleration; HMLD, high metabolic load distance; HR_max_, maximal heart rate; MRS, maximum running speed; SPR, average sprint distance; SPR_N, number of sprints; sRPE, session ratings of perceived exertion; TD, total distance; TL, Training load; TQR, total quality recovery; TRIMP, training impulse.

### External load measures

The ETL was tracked using a 18 Hz global positioning system (GPS) coupled with accelerometer (100 Hz), magnetometer (10 Hz) and gyroscope (100 Hz) (STATSports Apex^®^, Northern Ireland) (Buchheit et al., 2021). With a reliable satellite signal, all devices were turned on 30 min before the training data collection (Beato et al., 2018; Buchheit et al., 2021). The accuracy of GPS Apex^®^ devices was good (bias 5%) (Beato et al., 2018). The ETL measures were as follows: TD covered (m), average speed (AvS (m·min^−1^)), maximal running speed (MRS (m·s^−1^)), relative high-speed running (rHSR (m): 19.8–25.1 km·h^−1^) distance (m), high metabolic load distance (HMLD (m) > 25.5 W·kg^−1^), number sprints (*n*) and average sprint distance (SPR (m) (>25.1 km·h^−1^)) (m), dynamic stress load (DSL (a.u.)), number of ACC (>3 m·s^−2^) and number of decelerations (DEC < 3 m·s^−2^) (Teixeira et al., 2021b, 2022a) (Table 3).

### Internal training load measures

The ITL were obtained by RPE, TQR, and the HR monitors. A Garmin^®^ TM HR band (Garmin Ltd^®^, International Ltd., Olathe, KS, USA) was used to capture HR-based measurements utilizing a 1 Hz short-range telemetry system (Gómez-Carmona et al., 2020). Maximum heart rate (HR_max_), average heart rate (HR_mean_), percentage of HR_max_ (%HR_max_) and individual players’ training impulse (TRIMP) were monitored (Akubat et al., 2012; Branquinho, Ferraz & Marques, 2021). The Yo-Yo intermittent recovery test level 1 (YYIR1) was used to determine HR_max_ (Bangsbo, Iaia & Krustrup, 2008). The 15-point Portuguese Borg’s RPE 6-20 scale (Cabral et al., 2020) and TQR 6-20 score (Brink et al., 2010; Kenttä & Hassmén, 1998) were used to evaluate perceived effort. The entire time of training sessions for each participant was multiplied to get the session RPE (sRPE = RPE × session duration). Individual RPE’s and TQR’s were taken 30 min after and before each training session, respectively. Players were already familiarized with the RPE procedures by reporting in a Microsoft Excel^®^ spreadsheet (Microsoft Corporation^®^, Redmond, WA, USA) (Teixeira et al., 2021b, 2022a) (Table 3).

### Baseline characteristics

Players’ individual characteristics were collected by height (m), weight (kg), chronological age (years), sitting height (cm) and experience level (years). Anthropometric measures were measured using standard the International Society for the Advancement of Kinanthropometry (ISAK) guidelines (Marfell-Jones et al., 2006). Body mass (kg) was evaluated by an electronic scale Tanita MC 780-P MA^®^ (Tanita Corporation, Tokyo, Japan) with minimum clothing. Height (cm) was collected using an electronic stadiometer (Seca, Hamburg, Germany). Players’ height (m), weight (kg) and sitting height (cm) were recorded by the average of three measurements to the nearest 0.1 using international units (IU). Body mass index (BMI) was calculated by dividing weight by the square of height (kg/m^2^). BMI’s cut-offs used were: underweight < 18.5 kg/m^2^, normal 18.50–24.99 kg/m^2^, overweight ≥ 25 kg/m^2^, obese ≥ 30 kg/m^2^ (Suarez-Arrones et al., 2018). Relative age (a.u.) was calculated as the difference between the player’s birthdate and the cut-off date (31st August) was divided by the number of 365 days a year (Hill et al., 2020). Based on a predictive set of Mirwald’s equations, maturity offset and peak high velocity (PHV) were calculated (Mirwald et al., 2002; Teixeira et al., 2022a). Sub-elite young football was divided into pre-PHV (*n* = 52), mid-PHV (*n* = 65) and post-PHV (*n* = 207).

### Resultant equations for training load monitoring

The individual-based principal component in the resultant equations for TL monitoring were: low-moderate volume, high intensity, explosiveness, change of direction, collisions and body impacts (Rico-González et al., 2022a; Sheehan et al., 2020; Teixeira et al., 2021a). Also, the resultant equations added the baseline characteristics (*i.e*., anthropometric and maturational status) and the ITL measures (de Dios-Álvarez et al., 2021; Suarez-Arrones et al., 2018). Thus, the resultant equations was computed by a PCA approach can be expressed by the following algorithm (Jolliffe & Cadima, 2016):

$$PC{A_n} = \sum {\rm{ }}\Phi {i_1} \times {x_i} + {\rm{ }}\Phi {i_2} \times {x_2}\left(  \ldots  \right)\Phi {i_n} \times {x_n}$$

where the PCA_n_ is the *n* principal component, Φ is the loading vector comprising loadings (*i*_1_, *i*_1_…) of the first principal component. The loadings must have a sum of squares of exactly one. This is due to the possibility of a considerable variation when loadings are of a great magnitude.

It also specifies how the major component will move (PCA_n_), along which data varies the most (Jokiniemi, Pietilä & Mikkonen, 2021). The outcome is a line that is closest to the *n* observations in p-dimensional space. Euclidean distance squared is used to gauge proximity; x_n_ are normalized predictors. Normalized predictors (*x*_n_) have mean values equal to zero and standard deviations equal to one (Jokiniemi, Pietilä & Mikkonen, 2021; Jolliffe & Cadima, 2016). Resultant equation to quantify the weighted TL was expressed by:

$$T{L_{weekly}} = \sum P C{A_1} + PC{A_2}\left(  \ldots  \right)PC{A_n}$$

where the TL_Weekly_ is the sum of each PCA (p) and its weighted load vector (Jolliffe & Cadima, 2016).

### Statistical analysis

A data reduction technique was conducted using a principal component analysis (PCA) with 95% confidence intervals (95% CI) (Pino-Ortega et al., 2021; Rojas-Valverde et al., 2020). Monte Carlo parallel analysis were conducted to determine the number of extracted factors (Jokiniemi, Pietilä & Mikkonen, 2021). Z score were computed to scaled and centered final selection variables for PCA using Kaiser–Meyer–Olkin (KMO) values for measure of sampling adequacy and the Bartlett Sphericity test to ensure the sampled training data was suitable for data reduction. Factor analysis was acceptable when KMO values are greater than 0.6 and Bartlett Sphericity less than 0.05 (Pino-Ortega et al., 2021). The number of PCA to be retained was determined using the scree plot for the derived factor eigenvalues, considering eigenvalues greater than 1 (Rojas-Valverde et al., 2020). Factor’s components loading was computed using an orthogonal rotation with a VariMax method due to perpendicularity in the correlation matrix of the interest variables (Warmenhoven et al., 2019). Selection criteria for extraction of non-correlated variables was performed in *r* < 0.4 (Rojas-Valverde et al., 2020). Weightings (eigenvectors) are represented by a 2D plot and the results of the PCA are presented in a path analysis. The sample size was calculated by G*Power, Version 3.1.5.1 (Institut für Experimentelle Psychologie, Düsseldorf, Germany) with an effect size ß of 0.4, an α of 0.05, and a power of 0.8 (1−ß) (Teixeira et al., 2022a). Kolmogorov–Smirnov and Levene’s test were used to assess the normality and homogeneity. Statistical significance was set at *p* < 0.05. Data are presented as the mean ± SD using JASP software (JASP Team, 2022; jasp-stats.org).

## Results

### Data-reduction procedure, eigenvalue and component number

**Figure 2:**
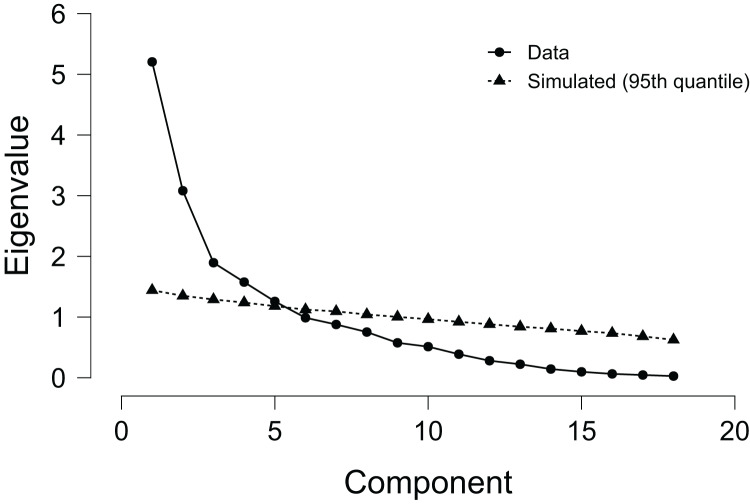
Scree plot for principal component analysis representing the component, explained variance and eigenvalues.

Figure 2 presents the eigenvalue ranged between 1.44% and 5.21%. Overall, five PCA accounted for 68.6% of the total explained variance. The five extracted PCA explained 27.4%, 16.2%, 10.0%, 8.3% and 6.7% of the variance in TL dataset, respectively. Thus, the first PC explained 27.4% of the TL by TD, HMLD, DSL, ACC and DEC. The second PCA explained 16.2% of the TL thought HSRr and SPR. The thirty PCA explained 10.0% of the TL *via* HR_max_, AvHR, %HR and TRIMP. The fourth PCA explained 8.3% of the baseline outset (*i.e*., sRPE, TQR, maturation offset and chronological age). The fifth PCA explained 6.7% of the accumulated TL (*i.e*., AvS and MRS). Constantly, PHV, relative age, experience level and BMI were excluded from the PCA (*r* < 0.4).

Table 4 also shows the data-reduction procedure resulting from rotated component matrix for accumulated TL variables with factor component loadings (eigenvectors). Four variables were excluded from the PCA due to the communalities below 0.4 (*i.e*., PHV, relative age, experience level and BMI). Also, KMO’s criteria reported a sampling adequacy of sampled data, reporting a considerable proportion of the variance as result of the underlying factors (KMO = 0.73). Furthermore, significant Barlett Sphericity test was significant (*p* < 0.001).

**Table 4:** Principal component analysis: data reduction procedure using varimax for rotated component matrix with factor loadings (eigenvectors) >0.4.

Variables	PC1	PC2	PC3	PC4	PC5	Uniqueness
TD (m)	0.698					0.365
AvS (m·min^−1^)					0.680	0.321
MRS (m·s^−1^)					0.790	0.259
HSRr (m)		0.928				0.041
HMLD (m)	0.788	0.501				0.123
SPR (n)		0.895				0.088
SPR (m)		0.940				0.066
DSL (au)	0.705					0.465
ACC (m·s^−2^)	0.844					0.233
DEC (m·s^−2^)	0.877					0.184
HR_max_ (bpm)			0.763			0.366
HR_Av_ (bpm)			0.967			0.055
%HR_max_			0.953			0.081
TRIMP (au)			0.692			0.501
sRPE (au)				−0.516		0.629
TQR (au)				−0.553		0.676
OFFSET (y)				0.669		0.343
Age (y)				0.836		0.261

**Note:**

Abbreviations: ACC, acceleration; AvHR, average heart rate; AvS, average speed; DEC, deceleration; HMLD, high metabolic load distance; HR_max_, maximal heart rate; MRS, maximum running speed; SPR, average sprint distance; SPR_N, number of sprints; sRPE, session ratings of perceived exertion; TD, total distance; TQR, total quality recovery; TRIMP, training impulse.

### Resultant equations and paths from principal components analysis

**Figure 3:**
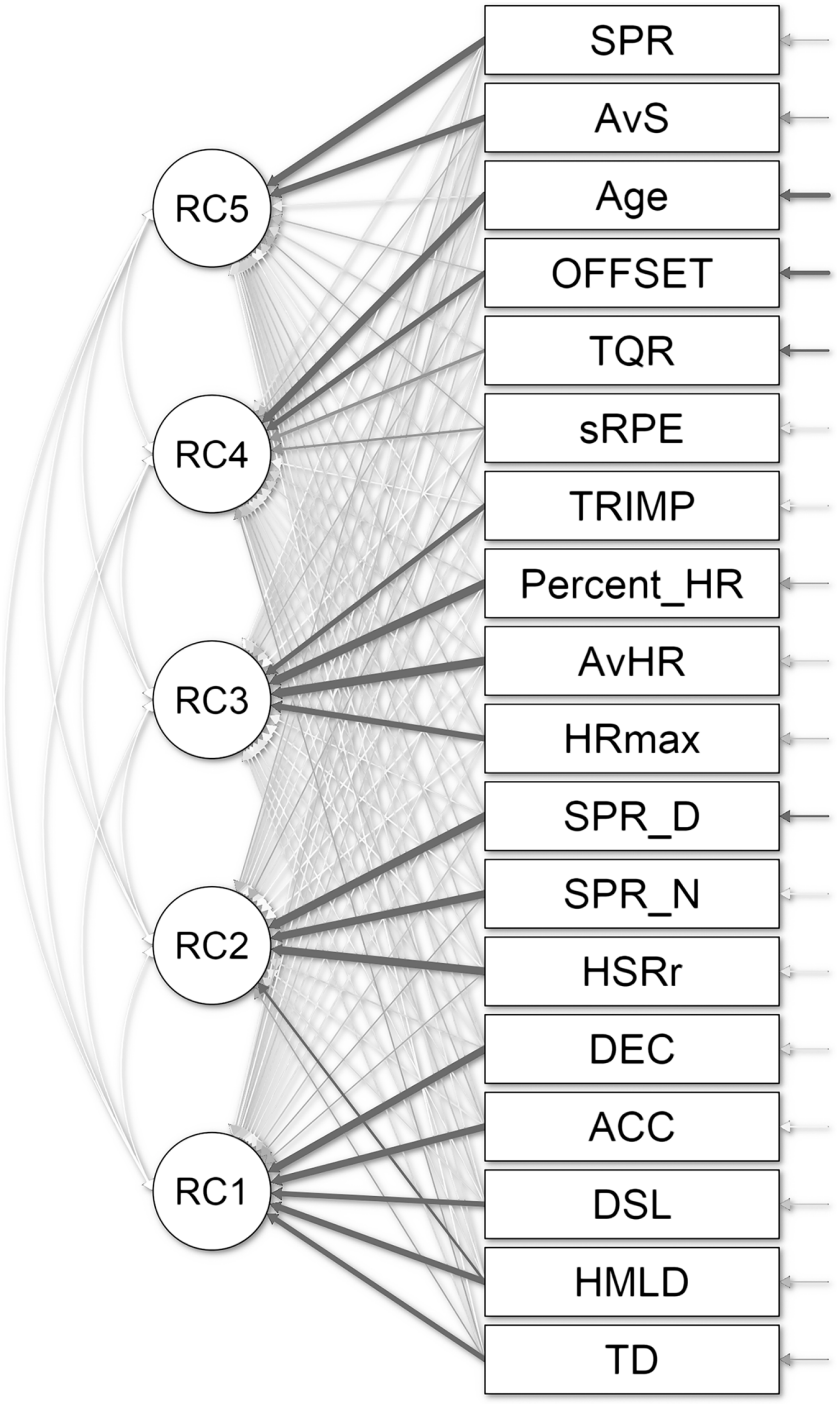
Principal component analysis and weightings (eigenvectors) were presented with a path.

The weightings (eigenvectors) of the PCA analysis are represented by a path graph in Fig. 3. Overall, the weightings ranged between −0.52 to 0.97. The highest weightings were observed in AvHR (bpm) (PCA 3) and the lowest weightings in sRPE (au) (PCA 4). Considering the highest factor in each principal component, the variables considered were TD (0.698), SPR (0.940), AvHR (0.967), Age (0.836) and MRS (0.790) for PCA 1 to PCA 5.

The resultant equations from extracted principal component are presented in Table 5. On this basis, the resultant equations for TL monitoring during a weekly microcycle can be expressed into five principal components determine the equations for the baseline variables: (1) explosiveness and impacts; (2) HSR; (3) HR measures; (4) baseline characteristics; (5) average running velocity.

**Table 5:** Resultant equations from extracted principal component analysis.

PCA	Construct	Variables	Calculation
1	Explosiveness, accelerations and impacts	TD (m), HMLD (m), DSL (au), ACC (>3 m·s^−2^), DEC (<3 m·s^−2^)	0.698 × TD (m) + 0.788 × HMLD (m) + 0.705 × DSL (au) + 0.844 × ACC (m·s^−2^) + 0.877 × DEC (m·s^−2^)
2	High intensity running	rHSR (19.8–25.1 km · h^−1^), SPR (*n*), SPR (m)	0.928 × rHSR (km · h^−1^) + 0.895 × SPR (*n*) + 0.940 × SPR (m)
3	Heart rate	HR_max_ (bpm), AvHR (bpm), %HR_max_, TRIMP (au)	0.763 × HR_max_ (bpm) + 0.967 × AvHR (bpm) + 0.953 × %HR_max_ + 0.692 × AkubatTRIMP (au)
4	Baseline characteristics	TQR (au), sRPE (au), Offset (y), Age (y)	−0.553 × TQR (au) + −0.516 × sRPE (au) + 0.669 × Offset (y) + 0.836 × Age (y)
5	Average running velocity	AvS (m·min^−1^), MRS (m·s^−1^)	0.680 × AvS (m · min^−1^) + 0.790 × MRS (m·s^−1^)

**Note:**

Abbreviations: ACC, acceleration; AvHR, average heart rate; AvS, average speed; DEC, deceleration; HMLD, high metabolic load distance; HR_max_, maximal heart rate; MRS, maximum running speed; SPR, average sprint distance; SPR_N, number of sprints; sRPE, session ratings of perceived exertion; TD, total distance; TQR, total quality recovery; TRIMP, training impulse.

## Discussion

The aim of this study was to reduce the dimensionality of the internal and external load measures, by a PCA approach, in order to describe and explain the resultant equations for TL monitoring during a standard microcycle in a sub-elite youth football players. Additionally, it is intended to identify the most representative measure for each principal component. After data reduction, five principal components were extracted from TL dataset explaining 68.7% of the total variance. The TL measures with the highest weight in each PCA were DEC, SPR distance, average HR, chronological age and MRS.

Resultant equations for TL monitoring during a standard microcycle in sub-elite youth football was split into: (1) explosiveness, ACC and impacts (27.4%); (2) HSR (16.2%); (3) heart bate-based measures (10.0%); (4) baseline characteristics (8.3%); (5) average running velocity (6.7%). Considering the highest representative factor in each principal component, the variables considered were DEC (PCA 1), SPR distance (PCA 2), average HR (PCA 3), chronological age (PCA 4) and MRS (PCA 5). In football, Pino-Ortega et al. (2021) also determined conditional dimensions such as angular velocity, speed displacements, HMLD, HSR, SPR, TD covered, metabolic power, DSL, jumps, impacts, ACC and DEC. The first PCA complies TD, HMLD, DSL, ACC and DEC, being grouped as explosiveness, ACC and impacts. Although there is a definite correlation between body impacts, ACC, and DEC. Otherwise, the TD may be due to an inverse relationship between training volume and intensity (Castillo et al., 2020). Also, the metabolic power was rather than speed-based zones to express running intensity (Osgnach et al., 2010). Nevertheless, the TD could fall outside this construct at first sight. An interaction effect between TD and DEC had already been documented for sub-elite football players (Teixeira et al., 2021b). The second PCA extracted HSRr and SPR, wherefore the HSR is an excellent variable to give meaning about training intensity (Harper et al., 2020). Zurutuza et al. (2020) combined peak velocity and distance covered at different velocities in the same principal component, confirming our results on high intensity demands. The third PCA complied the HR-based measures (*i.e*., HR_max_, AvHR, %HR_max_ and TRIMP), confirming the correlation between HR-based measures and ETL outcomes (de Dios-Álvarez et al., 2021; Ellis et al., 2021). The fourth PCA was explained by TQR, sRPE, maturation offset and chronological age. Although the fourth PCA has a lower variance explained it is fundamental to consider the influence of chronological age, biological age and perceived exertion (Teixeira et al., 2022a). In line with this component, the perceived exertion seems to be better explained with trainability, maturation and stage of development (Malina et al., 2019). Also, the TL could be influenced by acute: chronic workload ratio, training monotony and well-being variations (Clemente et al., 2021a, 2021b; Rico-González et al., 2022c). Indeed, the literature reported that greater acute: chronic workload ratio and training monotony levels are normally associated with an increased risk of injury or health issues. These measurements might be utilized to comprehend how the data changes throughout in-season phases (Rico-González et al., 2022a). Effectively, perceived exertion in young football players may be also influenced psychophysiological determinants as self-perception of competence and practice experience (Branquinho et al., 2021; Ferraz et al., 2017, 2018). Leading biological maturation in youth sports has become a research-practice gap still lacking knowledge about sub-elite environments using data reduction approaches (Cumming, 2018; Teixeira et al., 2021b, 2022c). Finally, the fifth PCA explained 6.7% of the accumulated TL thought AvS and MRS. Pacing behavior was also reported as a key point to football performance (Ferraz et al., 2018, 2020).

Research findings was slightly small than previous research in futsal (Rico-González et al., 2022a), Australian football (Sheehan et al., 2020), rugby (Scantlebury et al., 2020; Weaving et al., 2020) and Gaelic football (Gamble et al., 2019). Wherefore, the comparisons with current research would consider the differences between football codes. Scantlebury et al. (2020) reported a cumulative explained variance of 91%, 96% and 91% variance in TL in rugby union, field hockey and soccer. Casamichana et al. (2019) reported an explained variance of the external training intensity between 39% and 44%. Also, the eigenvalue of this study ranged between 1.44% to 5.21% by setting up values of accumulated TL substantially lower compared to other studies (*i.e*., eigenvalues between 1.0% and 68.0%) (Pino-Ortega et al., 2021; Scantlebury et al., 2020). Albeit, current research represents the first time that this statistical approach has been used in a sub-elite youth football, specifically using training data (Rico-González et al., 2022b; Rojas-Valverde et al., 2020).

Current applied PCA determine the resultant equations from individual-based principal components, expressing by major component weightings (Rico-González et al., 2022a; Sheehan et al., 2020; Teixeira et al., 2021a). Indeed, this is the traditional PCA algorithm that computes the principal components based on the covariance matrix or the singular value decomposition the data. It is widely used methods in team sports for dimensionality reduction, data visualization, and feature extraction (Pino-Ortega et al., 2021; Rico-González et al., 2022b; Rojas-Valverde et al., 2020). Other ratios, scores and equivalent equations have already been proposed to measure the TL, by emphasizing training intensity, volume or locomotion profile (Clemente et al., 2019; Owen et al., 2017; Rago et al., 2019). However, the PCA algorithms are diverse and some have not yet been implemented in football (Rico-González et al., 2022b; Rojas-Valverde et al., 2020). Hence, future perspective can explore other PCA algorithms such as incremental, Kernel, sparse and robust PCA approaches (Rojas-Valverde et al., 2020). Incremental PCA allows for incremental updates to the principal components as new data points are added in large datasets or when new data is continuously acquired, such as in real-time monitoring of football players’ performance or training data (Jokiniemi, Pietilä & Mikkonen, 2021). Kernel, sparse and robust PCA has been mainly applied for nonlinear dimensionality reduction, sparsity constraints and noisy or incomplete data (Teixeira et al., 2022c).

Futures research should expand the resultant equations by considering other well-being, technical-tactical and match-related contextual factors. Also, PCA approach must also consider the principal component in TL monitoring when considering training mode (*i.e*., small-sided and conditioned games), training day (*i.e*., MD-3, MD-2, and MD-1), age group (*i.e*., U15, U17, and U19) and maturational bands (*i.e*., pre-, mid- and post-PHV) (Teixeira et al., 2021a). Additionally, the training data represents only a specific sub-elite football academy and must be considered carefully when applied to another to other teams and contexts. As study limitations, the sample size and number of factors was rather small than previous studies with longer monitoring period (Rojas-Valverde et al., 2020). Also, the total variance was also relatively smaller for this PCA paths than other reports in football codes (Pino-Ortega et al., 2021; Rojas-Valverde et al., 2020). However, it must be ensured that football had the lowest percentage of the variance comparing with other football codes (Rojas-Valverde et al., 2020). Furthermore, choosing a higher threshold for total variance (%) may result in fewer retained principal components and a higher degree of data reduction with a consequent loss, noise or redundant information (Jokiniemi, Pietilä & Mikkonen, 2021; Jolliffe & Cadima, 2016). In general, there is no strict rule for the minimum value for percentage of total variance in PCA, as it depends on the specific application and the trade-off between data reduction and information retention (Rojas-Valverde et al., 2020). Furthermore, a commonly used threshold for retaining a principal component is to choose those components that explain at least 60–80% of the total variance, depending on the specific data analysis requirements (Jokiniemi, Pietilä & Mikkonen, 2021; Jolliffe & Cadima, 2016). Finally, the TL strategies applied in this quasi-experimental approach for only compiles GPS, HR and perceived exertion, however more objective measure of fatigue and recovery should be considered in futures reports, such as HR variability, electromyography signal intensity, biochemical markers and other well-being measures (Clemente et al., 2021a, 2021b). Also, further PCA approaches are needed to consider the principal components when integrating physical, physiological and tactical factors in football under an integrative perspective (Teixeira et al., 2022c).

## Practical applications

- Current resultant composite equations can be applied to relative contribution of the ITL and ETL measures for monitoring and management load in sub-elite youth football.
- Data reduction techniques decrease the redundant information and dimensionality of the training data, expressing in the following principal components: explosiveness and impacts, high-speed running, heart bate-based measures, baseline characteristics and average running velocity.
- Considering the highest factor in each principal component, DEC (PCA 1), sprint distance (PCA 2), average HR (PCA 3), chronological age (PCA 4) and maximal speed (PCA 5) are the conditional dimension to be considered in TL monitoring during a standard microcycle in sub-elite youth football players.
- Maturational status should be carefully considered in the TL monitoring together with relative age effect, chronological and baseline characteristics.
- Self-perception and practice experience may affect the variance explained by perceived exertion and pacing behavior.
- Training intensity and volume can be more accurately measured by current resultant composite equations and/or most representative factor for a standard microcycle in sub-elite youth football players.
- Futures research should expand the resultant equations for TL monitoring in sub-elite youth football with well-being, technical-tactical and match-related contextual factors.

## Conclusion

Using a PCA approach, five principal components could be applied to extract to describe and explain resultant equations for TL monitoring during an in-season standard microcycle in sub-elite youth football. Current research provides the first composite equations to extract the TL in this specific population expressed as explosiveness and impacts, high-speed running, HR-based measures, baseline characteristics and average running velocity. Considering the highest factor in each principal component, DEC (PCA 1), SPR distance (PCA 2), average HR (PCA 3), chronological age (PCA 4) and maximal SPR (PCA 5) are the conditional dimension to be considered in TL monitoring during a standard microcycle in sub-elite youth football players.

Future research should expand the resultant equations within the microcycle, by considering other well-being measures, technical-tactical factors and match-related contextual factors.

## Supplemental Information

10.7717/peerj.15806/supp-1 Supplemental Information 1 Raw Data. The individual data of the variables selected for this study.

## Additional Information and Declarations

### Data Availability

The following information was supplied regarding data availability: The raw measurements are available in the Supplemental File.

## References

[ref-1] Akubat I, Patel E, Barrett S, Abt G (2012). Methods of monitoring the training and match load and their relationship to changes in fitness in professional youth soccer players. Journal of Sports Sciences.

[ref-2] Bangsbo J, Iaia FM, Krustrup P (2008). The Yo-Yo intermittent recovery test: a useful tool for evaluation of physical performance in intermittent sports. Sports Medicine.

[ref-3] Beato M, Coratella G, Stiff A, Iacono AD (2018). The validity and between-unit variability of GNSS units (STATSports Apex 10 and 18 Hz) for measuring distance and peak speed in team sports. Frontiers in Physiology.

[ref-4] Bourdon P, Cardinale M, Murray A, Gastin P, Kellmann M, Varley M, Gabbett T, Coutts A, Burgess D, Gregson W, Cable N (2017). Monitoring athlete training loads: consensus statement. International Journal of Sports Physiology and Performance.

[ref-5] Branquinho L, Ferraz R, Marques MC (2021). 5-a-side game as a tool for the coach in soccer training. Strength & Conditioning Journal.

[ref-6] Branquinho L, Ferraz R, Travassos B, Marinho DA, Marques MC (2021). Effects of different recovery times on internal and external load during small-sided games in soccer. Sports Health.

[ref-7] Brink MS, Nederhof E, Visscher C, Schmikli SL, Lemmink KA (2010). Monitoring load, recovery, and performance in young elite soccer players. The Journal of Strength & Conditioning Research.

[ref-8] Buchheit M, Simpson BM, Hader K, Lacome M (2021). Occurrences of near-to-maximal speed-running bouts in elite soccer: insights for training prescription and injury mitigation. Science and Medicine in Football.

[ref-71] Cabral LL, Nakamura FY, Stefanello JMF, Pessoa LCV, Smirmaul BPC, Pereira G (2020). Initial validity and reliability of the Portuguese Borg rating of perceived exertion 6–20 scale. Measurement in Physical Education and Exercise Science.

[ref-9] Casamichana D, Castellano J, Díaz AG, Martín-García A (2019). Looking for complementary intensity variables in different training games in football. Journal of Strength and Conditioning Research.

[ref-10] Castillo D, Raya-González J, Clemente FM, Yanci J (2020). The influence of youth soccer players’ sprint performance on the different sided games’ external load using GPS devices. Research in Sports Medicine.

[ref-11] Clemente FM, Afonso J, Costa J, Oliveira R, Pino-Ortega J, Rico-González M (2021a). Relationships between sleep, athletic and match performance, training load, and injuries: a systematic review of soccer players. Healthcare.

[ref-12] Clemente FM, González-Fernández FT, Ceylan HI, Silva R, Younesi S, Chen YS, Badicu G, Wolański P, Murawska-Ciałowicz E (2021b). Blood biomarkers variations across the pre-season and interactions with training load: a study in professional soccer players. Journal of Clinical Medicine.

[ref-13] Clemente FM, Rabbani A, Conte D, Castillo D, Afonso J, Truman Clark CC, Nikolaidis PT, Rosemann T, Knechtle B (2019). Training/match external load ratios in professional soccer players: a full-season study. International Journal of Environmental Research and Public Health.

[ref-14] Coutinho D, Gonçalves B, Figueira B, Abade E, Marcelino R, Sampaio J (2015). Typical weekly workload of under 15, under 17, and under 19 elite Portuguese football players. Journal of Sports Sciences.

[ref-15] Cumming SP (2018). A game plan for growth: how football is leading the way in the consideration of biological maturation in young male athletes. Annals of Human Biology.

[ref-16] de Dios-Álvarez V, Suárez-Iglesias D, Bouzas-Rico S, Alkain P, González-Conde A, Ayán-Pérez C (2021). Relationships between RPE-derived internal training load parameters and GPS-based external training load variables in elite young soccer players. Research in Sports Medicine.

[ref-17] Ellis M, Penny R, Wright B, Noon M, Myers T, Akubat I (2021). The dose-response relationship between training-load measures and aerobic fitness in elite academy soccer players. Science and Medicine in Football.

[ref-18] Ferraz R, Gonçalves B, Coutinho D, Marinho DA, Sampaio J, Marques MC (2018). Pacing behaviour of players in team sports: influence of match status manipulation and task duration knowledge. PLOS ONE.

[ref-19] Ferraz R, Gonçalves B, Coutinho D, Oliveira R, Travassos B, Sampaio J, Marques MC (2020). Effects of knowing the task’s duration on soccer players’ positioning and pacing behaviour during small-sided games. International Journal of Environmental Research and Public Health.

[ref-20] Ferraz R, Gonçalves B, Tillaar R, Saiz S, Sampaio J, Marques M (2017). Effects of knowing the task duration on players’ pacing patterns during soccer small-sided games. Journal of Sports Sciences.

[ref-21] Ford PR, Bordonau JLD, Bonanno D, Tavares J, Groenendijk C, Fink C, Gualtieri D, Gregson W, Varley MC, Weston M, Lolli L, Platt D, Di Salvo V (2020). A survey of talent identification and development processes in the youth academies of professional soccer clubs from around the world. Journal of Sports Sciences.

[ref-22] Gamble D, Bradley J, McCarren A, Moyna NM (2019). Team performance indicators which differentiate between winning and losing in elite Gaelic football. International Journal of Performance Analysis in Sport.

[ref-25] Gómez-Carmona CD, Bastida-Castillo A, González-Custodio A, Olcina G, Pino-Ortega J (2020). Using an inertial device (WIMU PRO) to quantify neuromuscular load in running: reliability, convergent validity, and influence of type of surface and device location. The Journal of Strength & Conditioning Research.

[ref-23] Gonçalves B, Coutinho D, Exel J, Travassos B, Peñas C, Sampaio J (2019). Extracting spatial-temporal features that describe a team match demands when considering the effects of the quality of opposition in elite football. PLOS ONE.

[ref-24] Griffin A, Kenny IC, Comyns TM, Purtill H, Tiernan C, O’Shaughnessy E, Lyons M (2021). Training load monitoring in team sports: a practical approach to addressing missing data. Journal of Sports Sciences.

[ref-26] Haddad M, Stylianides G, Djaoui L, Dellal A, Chamari K (2017). Session-RPE method for training load monitoring: validity, ecological usefulness, and influencing factors. Frontiers in Neuroscience.

[ref-73] Harper DJ, Morin J-B, Carling C, Kiely J (2020). Measuring maximal horizontal deceleration ability using radar technology: reliability and sensitivity of kinematic and kinetic variables. Sports Biomechanics.

[ref-27] Hill M, Scott S, Malina RM, McGee D, Cumming SP (2020). Relative age and maturation selection biases in academy football. Journal of Sports Sciences.

[ref-28] Impellizzeri FM, Jeffries AC, Weisman A, Coutts AJ, McCall A, McLaren SJ, Kalkhoven J (2022). The ‘training load’ construct: why it is appropriate and scientific. Journal of Science and Medicine in Sport.

[ref-72] JASP Team (2022). JASP (Version 0.16.3) [Computer Software]. https://jasp-stats.org/download/.

[ref-29] Jokiniemi K, Pietilä AM, Mikkonen S (2021). Construct validity of clinical nurse specialist core competency scale: an exploratory factor analysis. Journal of Clinical Nursing.

[ref-30] Jolliffe IT, Cadima J (2016). Principal component analysis: a review and recent developments. Philosophical Transactions of the Royal Society A: Mathematical, Physical and Engineering Sciences.

[ref-31] Kenttä G, Hassmén P (1998). Overtraining and recovery: a conceptual model. Sports Medicine.

[ref-32] Los Arcos A, Mendiguchia J, Javier Y (2017). Specificity of jumping, acceleration and quick change of direction motor abilities in soccer players. Kinesiology.

[ref-33] Malina R, Cumming S, Rogol A, Coelho-e-Silva M, Figueiredo A, Konarski J, Koziel S (2019). Bio-banding in youth sports: background, concept, and application. Sports Medicine.

[ref-34] Marfell-Jones M, Olds T, Stewart A, Carter L (2006). ISAK accreditation handbook.

[ref-35] Mirwald RL, Baxter-Jones ADG, Bailey DA, Beunen GP (2002). An assessment of maturity from anthropometric measurements. Medicine and Science in Sports and Exercise.

[ref-36] Moura FA, Santana JE, Vieira NA, Santiago PRP, Cunha SA (2015). Analysis of soccer players’ positional variability during the 2012 UEFA European championship: a case study. Journal of Human Kinetics.

[ref-41] O’Donoghue P (2008). Principal components analysis in the selection of key performance indicators in sport. International Journal of Performance Analysis in Sport.

[ref-37] Oliva-Lozano JM, Muyor JM (2022). Understanding the FIFA quality performance reports for electronic performance and tracking systems: from science to practice. Science and Medicine in Football.

[ref-38] Oliva-Lozano JM, Rojas-Valverde D, Gómez-Carmona CD, Fortes V, Pino-Ortega J (2021). Impact of contextual variables on the representative external load profile of Spanish professional soccer match-play: a full season study. European Journal of Sport Science.

[ref-39] Osgnach C, Poser S, Bernardini R, Rinaldo R, Di Prampero PE (2010). Energy cost and metabolic power in elite soccer: a new match analysis approach. Medicine & Science in Sports & Exercise.

[ref-40] Owen AL, Djaoui L, Newton M, Malone S, Mendes B (2017). A contemporary multi-modal mechanical approach to training monitoring in elite professional soccer. Science and Medicine in Football.

[ref-42] Pino-Ortega J, Rojas-Valverde D, Gómez-Carmona CD, Rico-González M (2021). Training design, performance analysis, and talent identification—a systematic review about the most relevant variables through the principal component analysis in soccer, basketball, and rugby. International Journal of Environmental Research and Public Health.

[ref-43] Rago V, Brito J, Figueiredo P, Krustrup P, Rebelo A (2019). Relationship between external load and perceptual responses to training in professional football: effects of quantification method. Sports.

[ref-44] Rago V, Rebelo A, Krustrup P, Mohr M (2020). Contextual variables and training load throughout a competitive period in a top-level male soccer team. The Journal of Strength & Conditioning Research, Publish Ahead of Print.

[ref-45] Ric A, Torrents C, Gonçalves B, Sampaio J, Hristovski R (2016). Soft-assembled multilevel dynamics of tactical behaviors in soccer. Frontiers in Psychology.

[ref-46] Rico-González M, Oliveira R, González Fernández FT, Clemente FM (2022a). Acute: chronic workload ratio and training monotony variations over the season in youth soccer players: a systematic review. International Journal of Sports Science & Coaching.

[ref-47] Rico-González M, Pino-Ortega J, Praça GM, Clemente FM (2022b). Practical applications for designing soccer’ training tasks from multivariate data analysis: a systematic review emphasizing tactical training. Perceptual and Motor Skills.

[ref-48] Rico-González M, Puche-Ortuño D, Clemente FM, Aquino R, Pino-Ortega J (2022c). The most demanding exercise in different training tasks in professional female futsal: a mid-season study through principal component analysis. Healthcare.

[ref-49] Ricotti L, Rigosa J, Niosi A, Menciassi A (2013). Analysis of balance, rapidity, force and reaction times of soccer players at different levels of competition. PLOS ONE.

[ref-50] Rojas-Valverde D, Pino-Ortega J, Gómez-Carmona CD, Rico-González M (2020). A systematic review of methods and criteria standard proposal for the use of principal component analysis in team’s sports science. International Journal of Environmental Research and Public Health.

[ref-51] Ruan L, Ge H, Gómez MA, Shen Y, Gong B, Cui Y (2022). Analysis of defensive playing styles in the professional Chinese Football Super League ahead of print. Science and Medicine in Football.

[ref-52] Scantlebury S, Till K, Beggs C, Dalton-Barron N, Weaving D, Sawczuk T, Jones B (2020). Achieving a desired training intensity through the prescription of external training load variables in youth sport: more pieces to the puzzle required. Journal of Sports Sciences.

[ref-53] Sheehan WB, Tribolet R, Spurrs R, Fransen J, Novak AR, Watsford ML (2020). Simplifying the complexity of assessing physical performance in professional Australian football. Science and Medicine in Football.

[ref-54] Staunton CA, Abt G, Weaving D, Wundersitz DWT (2021). Misuse of the term ‘load’ in sport and exercise science. Journal of Science and Medicine in Sport.

[ref-55] Suarez-Arrones L, Petri C, Maldonado RA, Torreno N, Munguía-Izquierdo D, Di Salvo V, Méndez-Villanueva A (2018). Body fat assessment in elite soccer players: cross-validation of different field methods. Science and Medicine in Football.

[ref-56] Teixeira JE, Alves AR, Ferraz R, Forte P, Leal M, Ribeiro J, Silva AJ, Barbosa TM, Monteiro AM (2022a). Effects of chronological age, relative age, and maturation status on accumulated training load and perceived exertion in young sub-elite football players. Frontiers in Physiology.

[ref-57] Teixeira JE, Branquinho L, Ferraz R, Leal M, Silva AJ, Barbosa TM, Monteiro AM, Forte P (2022b). Weekly training load across a standard microcycle in a sub-elite youth football academy: a comparison between starters and non-starters. International Journal of Environmental Research and Public Health.

[ref-58] Teixeira JE, Forte P, Ferraz R, Branquinho L, Silva AJ, Monteiro AM, Barbosa TM (2022c). Integrating physical and tactical factors in football using positional data: a systematic review. PeerJ.

[ref-59] Teixeira JE, Forte P, Ferraz R, Leal M, Ribeiro J, Silva AJ, Barbosa TM, Monteiro AM (2021a). Monitoring accumulated training and match load in football: a systematic review. International Journal of Environmental Research and Public Health.

[ref-60] Teixeira JE, Forte P, Ferraz R, Leal M, Ribeiro J, Silva AJ, Barbosa TM, Monteiro AM (2021b). Quantifying sub-elite youth football weekly training load and recovery variation. Applied Sciences.

[ref-61] Teixeira JE, Forte P, Ferraz R, Leal M, Ribeiro J, Silva AJ, Barbosa TM, Monteiro AM (2022d). The association between external training load, perceived exertion and total quality recovery in sub-elite youth football. The Open Sports Sciences Journal.

[ref-62] Trecroci A, Milanović Z, Frontini M, Iaia FM, Alberti G (2018). Physical performance comparison between under 15 elite and sub-elite soccer players. Journal of Human Kinetics.

[ref-63] Trecroci A, Milanović Z, Rossi A, Broggi M, Formenti D, Alberti G (2016). Agility profile in sub-elite under-11 soccer players: is SAQ training adequate to improve sprint, change of direction speed and reactive agility performance?. Research in Sports Medicine.

[ref-64] Vanrenterghem J, Nedergaard NJ, Robinson MA, Drust B (2017). Training load monitoring in team sports: a novel framework separating physiological and biomechanical load-adaptation pathways. Sports Medicine.

[ref-65] Warmenhoven J, Cobley S, Draper C, Harrison A, Bargary N, Smith R (2019). Bivariate functional principal components analysis: considerations for use with multivariate movement signatures in sports biomechanics. Sports Biomechanics.

[ref-66] Weaving D, Beggs C, Dalton-Barron N, Jones B, Abt G (2019). Visualizing the complexity of the athlete-monitoring cycle through principal-component analysis. International Journal of Sports Physiology and Performance.

[ref-67] Weaving D, Dalton-Barron N, McLaren S, Scantlebury S, Cummins C, Roe G, Jones B, Beggs C, Abt G (2020). The relative contribution of training intensity and duration to daily measures of training load in professional rugby league and union. Journal of Sports Sciences.

[ref-68] Winter EM, Maughan RJ (2009). Requirements for ethics approvals. Journal of Sports Sciences.

[ref-69] Zurutuza U, Castellano J, Echeazarra I, Guridi I, Casamichana D (2020). Selection of training load measures to explain variability in football training games. Frontiers in Psychology.

